# Role of soil microbes in enhancing crop heterosis

**DOI:** 10.1002/imo2.20

**Published:** 2024-08-01

**Authors:** Yao Wang, Yong‐Fu Tao, Hong‐Ru Wang, Guo‐Hua Du, Qian‐Si Chen, Yong‐Xin Liu, Hong Lu

**Affiliations:** ^1^ Shenzhen Branch, Guangdong Laboratory of Lingnan Modern Agriculture, Genome Analysis Laboratory of the Ministry of Agriculture and Rural Affairs, Agricultural Genomics Institute at Shenzhen Chinese Academy of Agricultural Sciences Shenzhen China; ^2^ China Tobacco Gene Research Center, Zhengzhou Tobacco Research Institute of CNTC Zhengzhou China

## Abstract

Heterosis, or hybrid vigor, is characterized by the enhanced performance of F1 progeny in terms of yield, biomass, and environmental adaptation compared to their parental lines. Recent studies underscore the significant influence of soil microbes on heterosis, revealing that plant genotypes shape microbial communities which, in turn, have the potential to support plant growth through complex host‐microbe interactions. The deeper insight into microbial roles suggests innovative ways to boost crop performance and sustainability by managing the plant microbiome to further enhance heterosis.

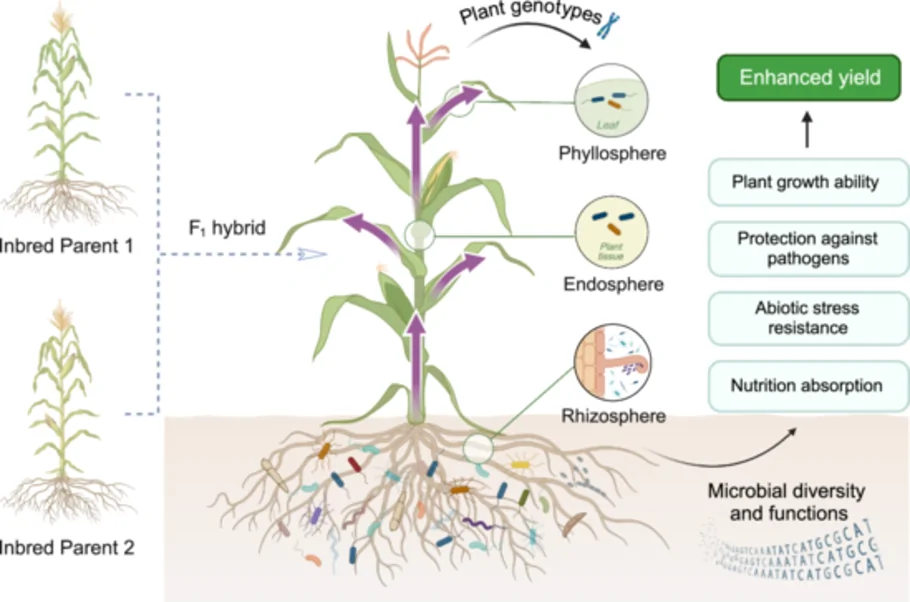

Heterosis, also known as hybrid vigor, refers to the superior performance on yield, biomass, and environmental adaption of the F_1_ progeny compared to their parental accessions [1]. Despite the significant yield improvement driven by heterosis in major crops, such as maize, rice, and sorghum, the molecular mechanisms underpinning heterosis remain largely elusive. Different hypotheses, such as dominance, overdominance, and epistasis, have been proposed to explain heterosis from the conventional perspective of quantitative genetics [2]. New evidence suggested that soil microbes, as the second genome of plants, play vital roles in the expression of heterosis. This work explores the intricate relationships between microbial communities and plant hosts that contribute to heterosis, offering insights into potential agricultural innovations for enhancing crop performance and sustainability.

Microbes, such as bacteria and fungi, inhibit various parts of plants, including the rhizosphere (soil around roots), phyllosphere (plant surfaces), and endosphere (interior tissues). The microorganisms establish complex interactions with plants, significantly influencing their growth, stress responses, and nutrient uptake [3]. These interactions potentially play a crucial role in the expression of heterosis, influencing the phenotypes of hybrid plants often resulting in improved growth and productivity over their inbred counterparts. This new perspective suggests that the plant genotype not only determines its phenotype but also shapes its microbial environment, which in turn, can enhance heterotic traits (Figure 1).

**Figure 1:**
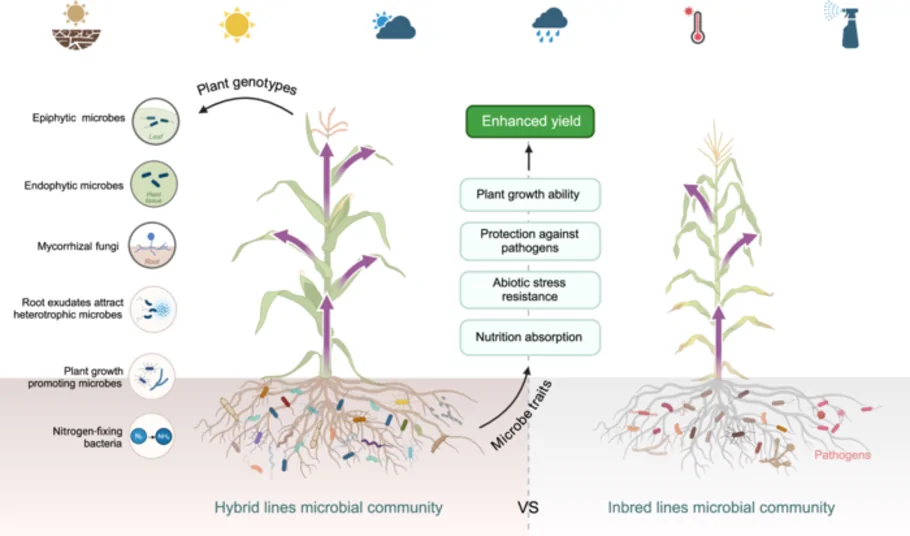
Comparison between the microbial communities associated with hybrid and inbred maize lines, emphasizing how host genotypes affect their associated microbial communities, which in turn affect the host's performance. The hybrid maize recruits a diverse microbial community contributing to enhanced yield. The inbred maize with a less diverse microbial community and pathogens may potentially reduce plant vigor and productivity. Microbial ecosystems play a role in promoting heterosis in hybrid plants, influenced by the interactions between host genotypes and their microbial partners, as well as environmental conditions.

## 1 INFLUENCE OF SOIL MICROBES ON HETEROSIS

Recent studies have highlighted the impact of plant genotypes on microbial community composition in their rhizosphere and phyllosphere, showing distinct differences between inbred lines and their F_1_ hybrids [4, 5]. For example, research on maize demonstrated significant variations in microbial communities associated with hybrids compared to inbred lines, suggesting that specific traits in hybrid plants potentially impact their microbiome composition [6]. Similar findings in rice have shown that hybrids host more diverse and abundant growth‐promoting microbes within their seeds than their parental lines. These beneficial microbes, such as those from the *Pseudomonas* and *Rhizobium* genera, could significantly enhance seed germination and early plant growth, providing a competitive advantage to hybrid plants [7].

Furthermore, microbial communities play a critical role in enhancing heterosis by impacting various plant traits [8]. For instance, in maize, soil microbes have been shown to influence root biomass and other traits associated with hybrid vigor. Notably, even under sterile conditions, where inbred lines perform comparably to their F_1_ offspring, heterosis can be restored by inoculation with a simple community of seven bacterial strains [9]. This finding highlights the importance of specific microbial communities in promoting plant growth and resilience. The study also revealed that the effect of microbes on heterosis varies with different environmental conditions. Different microbial compositions and environmental conditions can either enhance or suppress these beneficial effects, indicating a complex interaction between microbes, host plants, and their surrounding environment.

## 2 METHODOLOGICAL ADVANCES IN HETEROSIS STUDIES

**Figure 2:**
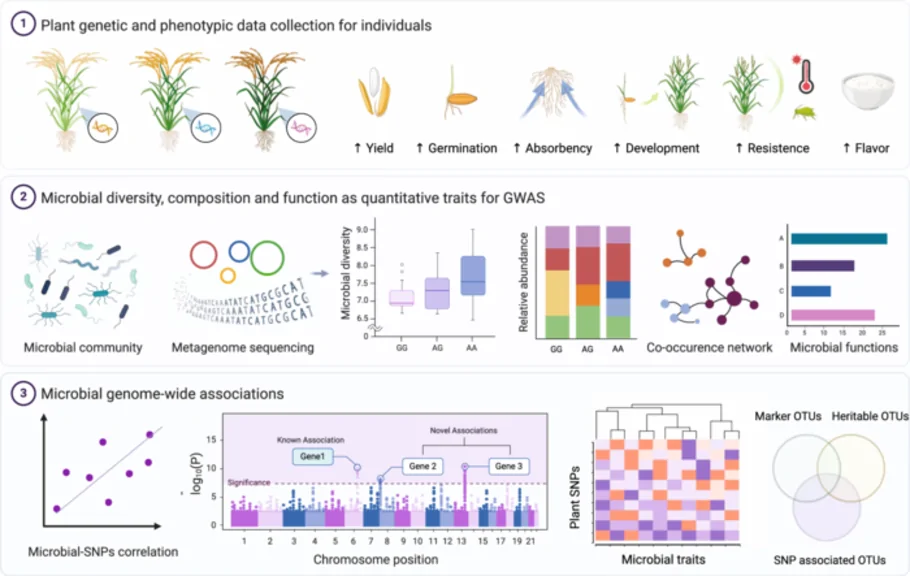
Integrated workflow of microbial genome‐wide association studies (mGWAS) in plants. Diverse plant genotypic and phenotypic data are collected to investigate how host genetic variations influence the associated microbial communities that contribute to heterosis. After sampling plant‐associated microbes and performing metagenomic sequencing, various microbial features such as diversity, abundance, and functional profiles are analyzed across different plant genotypes. These features are then selectively used as quantitative traits in mGWAS to elucidate how host genetic variations affect the assembly and function of the plant‐associated microbiota enhancing heterosis. OTU, operational taxonomic unit; SNP, single‐nucleotide polymorphism.

While the interaction between host genotype and the plant microbiome composition is presented, identifying the genetic factors that influence microbial community structure remains challenging. Advancements in genomic technologies have revolutionized the study of microbial communities associated with plants. Metagenomics, transcriptomics, and metabolomics allow for detailed characterization of these communities and help identify specific microbial species or functions linked to heterosis. Techniques such as microbial genome‐wide association studies (mGWAS), the use of microbial community composition as a quantitative trait, are particularly promising, providing insights into how plant genetic factors correlate with microbial community structures and functions [10] (Figure 2). Horton et al. [11] utilized phyllosphere microbial community data from *Arabidopsis thaliana* in a GWAS, revealing plant loci responsible for defense and cell wall integrity that significantly influence microbial community variation. Similarly, Deng et al. [12] revealed host genetic regulation in sorghum rhizosphere bacteria composition via mGWAS, offering new insights into the genetic factors that mediate microbial associations. A notable integrated study on 827 foxtail millet cultivars used GWAS, microbiome‐wide association studies (MWAS), and microbiome genome‐wide association studies (mGWAS) methods to unravel the associations between genotypic, phenotypic, and rhizoplane microbiota variables, identifying 257 rhizoplane microbial biomarkers associated with six key agronomic traits and validated the microbial‐mediated growth effects on foxtail millet using marker strains isolated from the field. The results demonstrated that microbial‐mediated growth effects on foxtail millet are dependent on the host genotype, suggesting that precision microbiome management could be strategically utilized to engineer high‐yielding cultivars in agricultural systems [13]. Joint analysis of metabolomic data set with microbiome data in these heterotic groups might be an effective approach in bridging the missing link through which microbiome affects the expression of heterosis.

The mechanisms of microbial influence on heterosis remain a subject of ongoing research, necessitating further elucidation. Specific interactions between plant genotypes and their associated microbiota are critical in addressing the problem [14]. These interactions may influence how microbial communities assemble and function, thereby affecting the expression of heterosis. Hybrids may recruit more diverse and functionally competent microbial communities than their parental lines. This enhanced microbial diversity is often correlated with improved nutrient acquisition and disease suppression, contributing to the observed heterosis. Microbial communities are known to produce a variety of metabolites that influence plant growth and stress responses [15]. Differences in the production of these metabolites between hybrids and their parental lines could be a factor contributing to heterosis. Moreover, microbes help plant defenses against both biotic and abiotic stresses [16, 17]. Hybrid might benefit from more effective microbial‐mediated stress responses, which could be part of the reason for their robustness compared to parental strains. The understanding of microbial contributions to heterosis has practical applications in agriculture. By leveraging beneficial microbial communities, it is possible to develop targeted inoculants or soil management practices that optimize the microbial environment for hybrid crops [18, 19]. This approach can enhance crop yields, improve resilience to environmental stresses, and reduce the need for chemical fertilizers and pesticides. Integrating microbial ecology with traditional breeding and genetic engineering offers a holistic approach to crop improvement, paving the way for more sustainable and productive agricultural systems.

## 3 CONCLUSION

In conclusion, the exploration of microbial contributions to heterosis represents a burgeoning field within agricultural science, offering promising avenues for enhancing crop performance. By deepening our understanding of how microbial communities interact with plant hosts to promote heterosis, researchers can develop innovative strategies that leverage the plant microbiome for agricultural benefits. Continued research in this area is likely to yield significant breakthroughs, paving the way for new sustainable agricultural practices via harnessing the potential of microbial management.

## AUTHOR CONTRIBUTIONS

**Yao Wang**: Writing—original draft. **Yong‐Fu Tao**: Writing—review and editing. **Hong‐Ru Wang**: Writing—review and editing. **Guo‐Hua Du**: Writing—review and editing. **Qian‐Si Chen**: Writing—review and editing. **Yong‐Xin Liu**: Conceptualization. **Hong Lu**: Conceptualization.

## CONFLICT OF INTEREST STATEMENT

The authors declare no conflict of interest.

## ETHICS STATEMENT

No animals or humans were involved in this study.

## Data Availability

No new data and scripts were generated in this commentary. Supplementary information (slides, videos, Chinese translated version, and updated materials) may be found in the online DOI or iMeta Science http://www.imeta.science/imetaomics/.
